# Reciprocal voltage sensor-to-pore coupling leads to potassium channel C-type inactivation

**DOI:** 10.1038/srep27562

**Published:** 2016-06-09

**Authors:** Luca Conti, Jakob Renhorn, Anders Gabrielsson, Fredrik Turesson, Sara I Liin, Erik Lindahl, Fredrik Elinder

**Affiliations:** 1 Department of Clinical and Experimental Medicine, Linköping University, Linköping, Sweden; 2 Theoretical and Computational Biophysics, Department of Theoretical Physics, KTH Royal Institute of Technology, Stockholm, Sweden; 3 Science for Life Laboratory, Department of Biochemistry and Biophysics, Stockholm University, Stockholm, Sweden

## Abstract

Voltage-gated potassium channels open at depolarized membrane voltages. A prolonged depolarization causes a rearrangement of the selectivity filter which terminates the conduction of ions – a process called slow or C-type inactivation. How structural rearrangements in the voltage-sensor domain (VSD) cause alteration in the selectivity filter, and vice versa, are not fully understood. We show that pulling the pore domain of the Shaker potassium channel towards the VSD by a Cd^2+^ bridge accelerates C-type inactivation. Molecular dynamics simulations show that such pulling widens the selectivity filter and disrupts the K^+^ coordination, a hallmark for C-type inactivation. An engineered Cd^2+^ bridge within the VSD also affect C-type inactivation. Conversely, a pore domain mutation affects VSD gating-charge movement. Finally, C-type inactivation is caused by the concerted action of distant amino acid residues in the pore domain. All together, these data suggest a reciprocal communication between the pore domain and the VSD in the extracellular portion of the channel.

Modulation of cellular excitability by altered function of voltage-gated ion channels plays a fundamental role in diseases such as epilepsy^1^, cardiac arrhythmias^2^, or episodic ataxia^3^. Following the voltage dependent opening most voltage-gated ion channels undergo a closure, called inactivation. In voltage-gated K^+^ (Kv) channels at least two types of inactivation have been described^4^: a fast (milliseconds) N-type inactivation and a slow (seconds) C-type inactivation. Deletion of the N-terminus eliminates the fast inactivation, and the slower C-type inactivation is revealed. C-type inactivation is associated with conformational alterations in two parts of the channel: (1) the selectivity filter at the center of the pore domain, consisting of transmembrane segments S5 and S6 from all four channel subunits^5^,^6^,^7^,^8^,^9^,^10^,^11^,^12^; and (2) the peripherally located voltage-sensor domains (VSDs) comprising segments S1 to S4 of each of the four subunits (Fig. 1a,b)^13^,^14^,^15^,^16^.

High concentrations of K^+^ decrease the rate of C-type inactivation^7^ while low concentrations make the channel non-functional, sometimes permanently^17^. When combined with the fact that ion flux is dependent on a close interplay between several K^+^ binding sites in the pore^18^,^19^, this suggests that loss of K^+^ from the selectivity filter destabilizes its structure enough to prevent conduction. Functional studies suggest that the selectivity filter widens during slow inactivation^6^,^20^. Such a widening is supported by structural studies showing that low K^+^ concentrations prevent K^+^ from being coordinated efficiently because of a conformational change in the selectivity filter^12^,^21^.

The voltage dependence of voltage-gated channels is mediated by the mobile charge-containing fourth transmembrane segment, S4, in the VSD^22^,^23^,^24^,^25^,^26^. Movement of this charged segment generates detectable gating currents^22^,^27^,^28^. However, the gating charge movement depends on the holding, or prepulse, voltage. A holding voltage around −80 mV keeps S4 in a resting (down) state even after depolarization to −40 mV, while a holding voltage around 0 mV keeps S4 in an activated (up) state after polarization to the same −40 mV. The gating charge vs. voltage, *Q*(*V*), curve thus depends on the holding voltage. Alteration in holding voltage can shift the *Q*(*V*) by up to 50 mV^13^. Such a *Q*(*V*) shift has been described for Na^+^ channels^29^, Kv channels^13^,^15^, pace-maker HCN channels^16^, and also isolated VSDs like the voltage-dependent phosphatase Ci-VSP^30^. This *Q*(*V*) shift has been associated with C-type inactivation^13^ but a direct communication between the pore domain and the VSD has not been demonstrated.

In this study we examine a possible molecular link between the VSDs and the pore domain, with respect to C-type inactivation. Previous studies have shown that the conserved glutamate at the extracellular end of S5 in the pore domain (E418 in the Shaker K channel) is necessary for the slow time-course of C-type inactivation – mutations to either cysteine^15^ or glutamine^31^ accelerates the inactivation drastically. A neighboring residue, F416, is in close contact with the VSD in an X-ray crystal structure^24^ (Fig. 1a,b) and has been implicated as a candidate for a functional VSD-to-pore domain connection^20^,^32^. Further, residues in the neighborhood of F416 form an important, close, and rigid contact point between the VSD and the pore domain^33^,^34^,^35^,^36^,^37^. Here we explore the hypothesis that residue F416 is a possible candidate in the reciprocal VSD-to-pore interaction that modulates C-type inactivation.

## Results

### Residue F416 is a remote player in C-type inactivation

**Figure 1:**
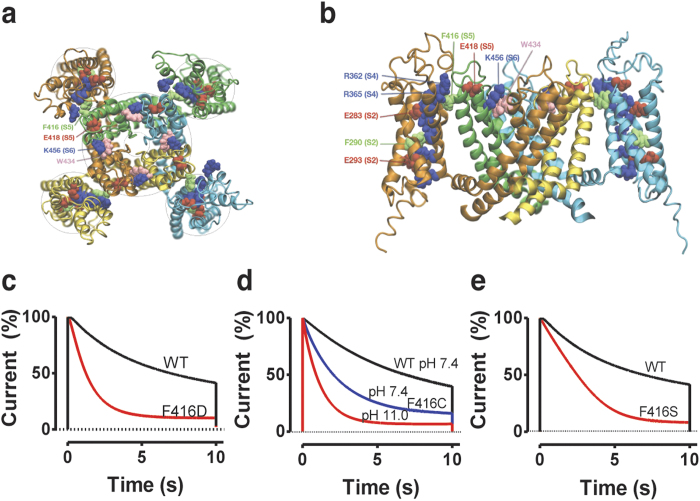
The pore domain/VSD interface is important for C-type inactivation. (**a**,**b**) Molecular structure of the Shaker Kv channel (top view in **a**, side view in **b**). Four identical voltage-sensor domains (VSDs) surround the pore domain. The voltage sensor S4 (positively charged residues in blue) is in an activated up-state. For clarity, the VSDs in the front and the back are removed (**b**). Labelled residues are discussed in this paper. (**c–e**) Effects of different mutations and pH on the inactivation time course and steady-state current. Test step voltage = +80 mV. Holding voltage = −80 mV. pH = 7.4 if not otherwise noted.

Substituting an aspartate for a phenylalanine at position 416 (F416D) significantly (*p* < 0.01) accelerated C-type inactivation, by a factor of 4.0 (Fig. 1c), from *τ* = 4.8 ± 0.1 s (*n* = 3) to 1.2 ± 0.2 s (*n* = 3) and reduced the steady-state current from 30 ± 2% (*n* = 3) to 8 ± 1% (*n* = 3) of the peak current at +80 mV. Switching from a low (1 mM) to a high (100 mM) K^+^ concentration in the extracellular solution slowed down the rate of inactivation at +50 mV by a factor of 2.6 ± 0.6 (*n* = 3), a hallmark for C-type inactivation and an alteration in the selectivity filter. Because aspartate is both charged/polar and relatively short, the faster inactivation could either be a consequence of the size and/or the charge. Neither the slightly longer and negatively charged amino-acid residue glutamate, nor the short but uncharged alanine had any effect on C-type inactivation (Supplementary Fig. S1a), suggesting that a short *and* a negatively charged/polar residue is required to accelerate C-type inactivation. To test this, we tried a cysteine, which is relatively short with a partial charge at physiological pH (p*K*a = 8.3). F416C at pH 7.4 significantly (*p* < 0.05) accelerated inactivation from *τ* = 4.8 ± 0.1 s (*n* = 3) to 3.2 ± 0.2 s (*n* = 5) and reduced the steady-state current from 30 ± 2% (*n* = 3) to 10 ± 1% (*n* = 5) of the peak current at +80 mV (Fig. 1d). F416C at pH 11, expected to result in a negative net charge of the cysteine, inactivated 4 times faster τ = 1.0 ± 0.2 s, *n* = 4) than wt at pH 11 *τ *= 3.6 ± 0.2 s; *n* = 4) (Fig. 1d). The short and polar F416S also accelerated the inactivation from *τ* = 4.8 ± 0.1 s (*n* = 3) to 2.4 ± 0.5 s (*n* = 3; *p* < 0.05) (Fig. 1e). In contrast, the polar but larger F416Q and the bulky F416W did not affect the inactivation (Supplementary Fig. S1a). The positively charged F416R and F416K did not express; to overcome this, we explored F416H in pH 6.0 (where histidines are expected to be positively charged), but the inactivation was not affected (Supplementary Fig. S1b). The effect on C-type inactivation is not correlated to any effect on the voltage-dependence of the opening of the channel (Supplementary Table I).

To conclude, a short and negatively charged or polar residue at 416 clearly accelerated C-type inactivation, while larger, nonpolar, or positively charged residues had no effect. Because F416 is close to the positive gating charges of S4 in the VSD we hypothesized that the negative charge of F416D is attracted to the gating charges, leading to distortion in the top of S5, altered interactions with S6, and eventually a change in geometry of the selectivity filter and C-type inactivation. The slightly longer glutamate (approximately 1 Å) did not cause this alteration, suggesting a well-defined connection between the VSD and the pore domain.

### Specific interactions between the pore domain and the VSD trigger C-type inactivation

If an electrostatic attraction of 416 towards the VSD accelerates C-type inactivation it should be possible to identify interacting partners by metal-ion bridges promoting C-type inactivation. Functional^34^,^38^ and structural^24^ studies show that F416 is close to the gating charge R365 in the open state. The neighboring residues A359, R362, and R368 are expected to sweep along the same path during the transition to the resting state; A359 and R362 are expected to pass close to 416 before reaching the open state, and R368 is, together with R365, close to 416 in the open state^25^,^39^.

**Figure 2:**
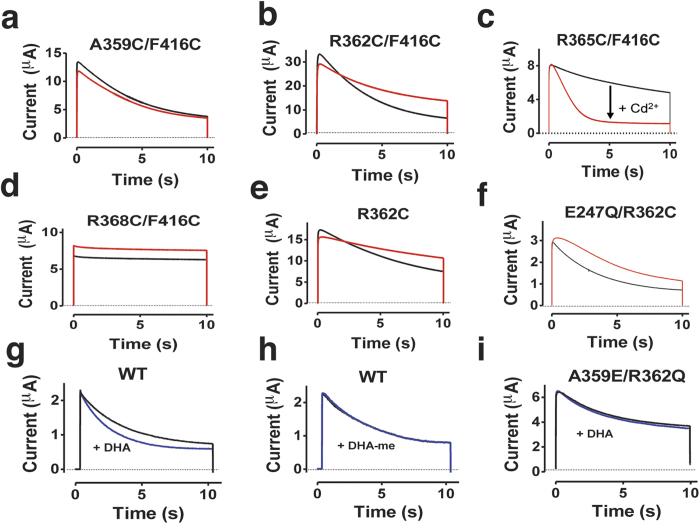
Interactions between F416C of S5 and residues of S4 affect C-type inactivation. (**a–e**) 10 μM Cd^2+^ (red) affects the time course of slow inactivation in R362C/F416C (**b**) and R365C/F416C (**c**) but not in R359C/F416C (**a**) or R368C/F416C (**d**). Control in black. (**e**) 10 μM Cd^2+^ (red) slows down inactivation in R362C single mutant. (**f**) 10 μM Cd^2+^ (red) does not affect the inactivation time constant in E247Q/R362C. (**g**) 70 μM DHA (blue) accelerates inactivation in Shaker wt. (**h**) 70 μM DHA-me (blue) does not accelerate inactivation in Shaker wt. (**i**) 70 μM DHA (blue) only has a minor effect on inactivation in A359E/R362Q. For all panels, test step voltage = +80 mV, holding voltage = −80 mV, pH = 7.4.

10 μM Cd^2+^ did not affect C-type inactivation of A359C/F416C at +80 mV (Fig. 2a). Cd^2+^ significantly (*p* < 0.05) slowed down C-type inactivation of R362C/F416C, from τ = 4.5 ± 0.2 s to τ = 7.8 ± 0.3 s (*n* = 3; Fig. 2b). Cd^2+^ significantly (*p* < 0.05) accelerated C-type inactivation of R365C/F416C, by a factor of 6, from τ = 7.1 ± 2.9 s to τ = 1.2 ± 0.1 s and reduced the steady-state current from 25 ± 9% to 8 ± 1% of the peak current at +80 mV (*n* = 3; Fig. 2c). Cd^2+^ had no effect on the time course of R368C/F416C (Fig. 2d). All single mutants were unaffected by Cd^2+^ (Supplementary Fig. S2a,b) except for R362C (Fig. 2e). Cd^2+^ slowed inactivation in R362C mutants (from τ = 6.5 ± 0.4 s to τ = 11.9 ± 0.2 s, *n* = 6) to a similar extent as in R362C/F416C, suggesting that the Cd^2+^ effect on R362C/F416C was probably not caused by a Cd^2+^ bridge between these two residues. Cd^2+^ can also form a metal-ion bridge between one cysteine and one glutamate^25^. R362C is nearby E247 in the last closed state, before the channel reaches the open state^25^. Thus, a Cd^2+^ bridge between E247 and R362C prevents, or slows down, the clockwise rotation of S4 that opens the channel. To test if a Cd^2+^ bridge between R362C and E247 is the reason for the slowing down of C-type inactivation we neutralized the charge of E247. Cd^2+^ had a minor effect on the amplitude of E247Q/R362C, but no effect on the inactivation time constant (Fig. 2f). Thus a Cd^2+^ bridge between E247 and R362C is the major cause of the slowed inactivation. In an attempt to create a specific interaction between residues 362 and 416 we tried to make Zn^2+^ bridges^40^ in R362H/F416H, but this channel did not express.

If a counter-clockwise rotation of S4 (when viewed from the extracellular side; to satisfy a E247E/R362C Cd^2+^ bridge) slows down inactivation, then we hypothesize that a clockwise rotation of S4 can accelerate C-type inactivation. To test this, we employed polyunsaturated fatty acids (PUFAs), that previously has been shown to rotate S4 clockwise^38^,^41^. 70 μM docosahexaenoic acid (DHA) clearly accelerated C-type inactivation; the time constant was reduced by 34 ± 4% (*n* = 6; *p* < 0.01; Fig. 2g). All these data are consistent with a rotation of S4 to open the channel, but another possibility is that S4 is more or less tilted and that the E247E/R362C-bridge and the DHA molecule tilts S4 in different directions. As a critical test of the hypothesis that the charge of DHA accelerates C-type inactivation by rotating S4 clockwise or by tilting S4 towards the lipid bilayer, we performed two types of experiments. First, methyl esters bind to the same position on K channels as PUFAs^42^ but are uncharged and thus should not rotate or tilt S4 to affect the C-type inactivation. We found that the inactivation time constant was not affected by 70 μM of DHA-methyl ester (0 ± 5%; *n* = 6; Fig. 2h). Second, we tested if a mutation with lower sensitivity to DHA (A359E/R362Q^41^) also reduced the DHA effect on C-type inactivation. Indeed, 70 μM DHA accelerated C-type inactivation significantly less in the A359E/R362Q channel compared to wt (−23 ± 3%; *n* = 7; *p* < 0.05; Fig. 2i). Thus, these data supports the hypothesis that the rotation and/or tilt of S4 due to the interaction with DHA affects C-type inactivation.

### A Cd^2+^ bridge within the VSD affects C-type inactivation

**Figure 3:**
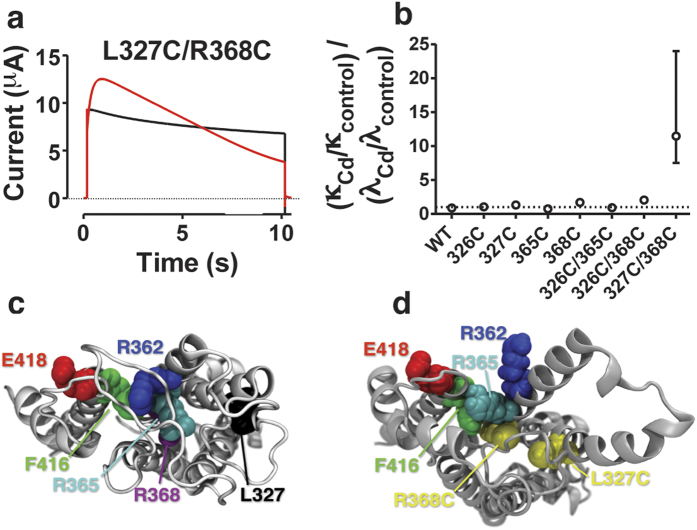
Interactions between S3 and S4 in the VSD affect C-type inactivation. (**a**) 10 μM Cd^2+^ (red) inactivates L327C/R368C to a lower steady-state current. Control in black. Test step voltage = +80 mV. Holding voltage = −80 mV. pH = 7.4. (**b**) Quotient of Cd^2+^ effects on the rate constants *λ* and *κ* calculated according to Scheme I (see Methods for details) for eight different channels. Error bars are calculated from the inverse of the data. *n* = 3–4. (**b**) Top view of model of the VSD and S5 of the Shaker channel based on a crystallographic structure. (**d**) Top view of the model based on our experimental data. Note how a Cd^2+^ bridge between L327C and R368C lifts R365 (cyan) above F416 (green) without moving more positive gating charges across the central hydrophobic barrier of the VSD.

If a direct interaction between the VSD and the pore domain affects C-type inactivation, alteration solely within the VSD could potentially alter the VSD structure and thereby the VSD-to-pore interaction and consequently the C-type inactivation. To guide our targeting of Cd^2+^ bridges within the VSD^25^ we identified several interactions in the open state that potentially could affect slow inactivation. Here we report that Cd^2+^ on L327C/R368C significantly (p < 0.05) decreased the steady-state current at +80 mV from 63 ± 3% to 12 ± 6% of peak current (*n* = 4; Fig. 3a). The inactivation rate was slightly accelerated and the peak current was increased even though the current was measured at a voltage where the *G*(*V*) curve had reached a flat level. Two other confirmed Cd^2+^ bridges, T326C/R365C and T326C/R368C did not affect C-type inactivation (Supplementary Fig. S3). Cd^2+^ did not affect the time constant of inactivation, the steady-state current, or the recovery from C-type inactivation of the wt channel or of the single mutants T326C, L327C, R368C, R365C (Supplementary Fig. S4 and S5), confirming that the Cd^2+^ effect on L327C/R368C was mediated by a Cd^2+^ bridge between these two residues.

To analyze the Cd^2+^ effect on inactivation in more detail we calculated the rate constants *κ* and *λ* assuming the following kinetic scheme (*C* = closed, *O* = open, *I* = inactivated; see Methods for calculations):


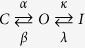


The major effect of Cd^2+^ on L327C/R368C was a decrease in *λ*, and a minor increase in *κ* (Supplementary Fig. S5), both effects promoting C-type inactivation of the channel. (Note that the rate λ is not equivalent to recovery from slow inactivation, so this rate could not be measured by a conventional two-pulse protocol). The alteration of the quotient *κ*/*λ* is a good measure of the effect on inactivation; Cd^2+^ increased *κ*/*λ* by a factor of 12 in L327C/R368C mutants, while all other studied single and double mutants were not affected (Fig. 3b).

What is the molecular mechanism of the effect of the L327C/R368C Cd^2+^-bridge? L327C and R368C are relatively distant from each other in the crystal structure (Fig. 3c), and a structural rearrangement is required to make L327C and R368C close enough to create a Cd^2+^ bridge. A Cd^2+^ bridge between L327C and R368C might compress the VSD, and a reduced “pressure” on the pore domain makes *λ* smaller. To get an idea of the required rearrangement, we constructed molecular models of the VSD by Rosetta modelling and molecular dynamics simulations (Fig. 3d). When the L327C/R368C Cd^2+^ bridge is formed by applying harmonic constraints to L327C/R368C to model the bridge in Rosetta, the gating charge R365 is lifted slightly above F416, leaving room for an expansion of the pore domain towards the VSD, but no extra gating charges were transferred through the central hydrophobic core of the VSD. This minor alteration can be the explanation for the effect on C-type inactivation.

### Molecular modelling of C-type inactivation

**Figure 4:**
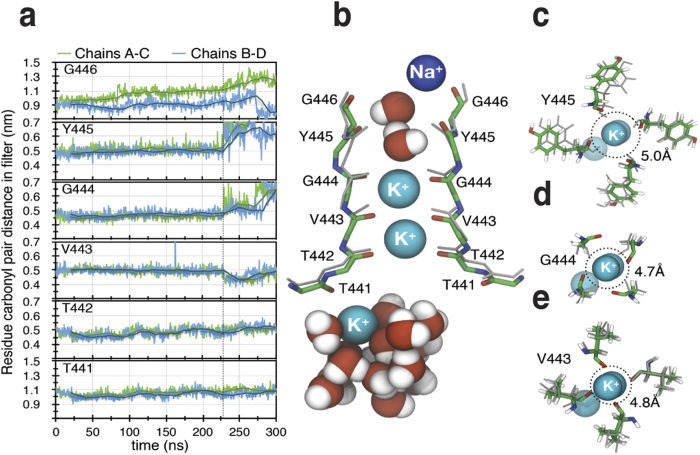
Molecular modelling of the mechanism for C-type inactivation. (**a**) Distances between the α carbons for six residues in the selectivity filter during a 300 ns long MD simulation when all four 416 residues are pulled away from the center of the channel at a rate of 0.001 nm/ns (see Methods for details). After 229 ns (vertical line) the distances are abruptly altered and the first K^+^ leaves the filter array. (**b**) A snapshot at the time of the filter collapse (229 ns) shows that the array of K^+^ in the selectivity filter retreat downwards. Water and Na^+^ interact with carbonyls (red sticks) in positions 445 and 446. Structure is aligned to initial frame of the simulation (grey sticks) with regard to N, CA, C, and O backbone atoms in position 441 to 446 of all four chains. (**c–e**) Filter distortion (top view). At positions 445 (**c**) and 444 (**d**) there is an increase in filter diameter (average of distances between carbonyls of opposing chains) compared to initial structure (grey sticks, dotted circle). At position 443 (**e**) there is a decrease in filter diameter. There is also side chain rotations of Y445 at the moment of filter collapse (**c**).

The experimental data presented so far are consistent with an hypothesis that pulling residue F416 towards the VSD leads to C-type inactivation. To test this hypothesis, that F416 has to move towards the VSD, to promote C-type inactivation, we used steered molecular dynamics simulations of a Shaker model embedded in a lipid bilayer (see Methods for details) to pull F416 away from the pore. Utilizing a pull coordinate defined as the center-of-mass-distance between F416 in opposing subunits avoided the issue of defining a reaction force group in the remaining portions of the channel, and any resulting torque. Pulling was achieved by introducing a potential to restrain the F416 pair distance to its initial value (using a force constant of 2000 kJ/mol/nm^2^, such that a 0.1 nm displacement from the reference position results in a force of 200 kJ/mol/nm) and the reference distance was then increased by 0.002 nm/ns during the simulation. This moves each F416 residue outwards from the pore in a direction similar the Cd^2+^ bridge (Supplementary Fig. S6) at an effective rate of roughly *half* this value (since the reference is measured between the two F416 residues), with some fluctuations. The extent of the displacement of F416 is compatible with a Cd^2+^ bridge between F416C and R365C, but it should be kept in mind the simulation is a model of the distortion in the pore rather than a model of metal-ion bridge rigidity. Four simulations of 300 ns each were performed, as well as a control simulation without any pull force. In all simulations except the control, the force on F416 caused a slight outward motion of S5, which was mediated to S6 through E418, which in turn caused a rearrangement of the side chain interactions between K456 and W434 just outside the selectivity filter (Fig. 1). For two out of the four simulations (#2 and #4), this led to a distinct perturbation of the selectivity filter characterized by shifts in the geometry of the backbone carbonyls in positions 443 through 445, which was immediately followed by K^+^ leaving the binding site in the filter (Fig. 4a,b) and likely making the channel non-conducting. In the upper part of the filter (Y445, G446), carbonyls were displaced outwards and upwards (Supplementary Fig. S7), which increased the diameter of the filter and destroyed the coordination of the ion binding site (Fig. 4c,d,e). As K^+^ dissociated, water and Na^+^ entered the filter. In the lower part of the filter (in vicinity of V443) the carbonyls were displaced downwards (i.e., an elongation of the entire filter; Supplementary Fig S7) with maintained ion coordination (Fig. 4b,e). In simulation #3 the distortion of the pore occurred slower (over 150 ns), but the end result was the same – complete dissociation of the K^+^ ion. Finally, in simulation #1, the same distortion and distance increase in the upper filter carbonyl geometry were observed, but in this case K^+^ had not dissociated after 300 ns. It is worth noting that the effect observed is not a plain outward motion in the same direction as the pulling, but mainly a gradual straining of the pore that leads to a filter extension. The control simulation showed no perturbation of the structure for the filter (average carbonyl-carbonyl displacement from initial structure in positions 443 to 445 smaller than 0.02 nm, with standard error 0.04 nm). A second control was performed by removing the pulling force after 225 ns, which resulted in the pore relaxing back (Supplementary Fig. S8).

### Concerted action in C-type inactivation

So far, both experimental and computational data in this study suggests that an interaction between F416 and the VSD lead to C-type inactivation. It is known that C-type inactivation is a cooperative process between the four subunits, involving residues at the center of the ion channel^7^,^8^,^9^,^43^ or residues more distant from the selectivity filter^15^. However, it is not known if residues in the periphery act in concert with residues at the center of the channel. To explore this we mutated non-interacting pairs of residues known to affect C-type inactivation, at the extracellular surface of the channel protein. If two residues act in concert, the energetics of the two mutations will be added, meaning that the alteration in the inactivation rate caused by the first mutation multiplied by the alteration caused by the second mutation should equal the alteration caused by the double mutant. If two residues act independent of each other, the inactivation rate of the double mutant should equal or close to the inactivation rate of the fastest single mutant (see Supplementary information for details).

**Figure 5:**
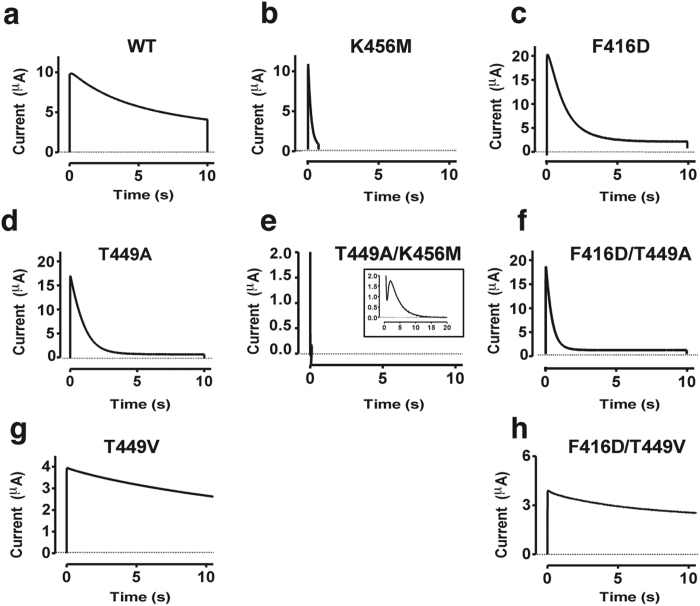
Concerted action within a subunits during C-type inactivation. (**a–h**) Inactivation of eight different channels as denoted in the panels. Test step voltage = +80 mV; holding voltage = −80 mV; pH = 7.4. An inset is shown for T449A/K456M for a 20 ms long pulse (**e**). The pulses for T449V (**g**) and F416D/T449V (**h**) were 60 s long but for comparative reasons only 10 s are shown.

We explored all possible combinations of four residues covering a large area of the extracellular surface of the pore domain to test if they act in concert (416, this study; 418^15^,^31^; 449^7^,^8^; 456, this study). 449 and 456 clearly acted in concert during C-type inactivation: T449A (Fig. 5a,d) and K456M (Fig. 5a,b) inactivated 13 and 20 times faster than wt respectively, and the double mutant T449A/K456M inactivated 1600 times faster than wt (Fig. 5a,e). Among all other tested combinations (F416D/E418C, F416D/K456M, F416D/T449A, F416D/T449V, E418C/K456M, and E418C/T449A), only F416D/T449A and F416D/T449V generated clearly recordable currents. F416D/T449A (Fig. 5f) inactivated clearly faster than the single mutants F416D (Fig. 5c) and T449A (Fig. 5d) suggesting a concerted action. F416D/T449V (Fig. 5h) inactivated at the same rate as wt, thus much slower than F416D (Fig. 5c) but faster than T449V (Fig. 5g), as expected for concerted action, and in sharp contrast to a non-concerted action.

### A pore domain mutation affects gating charge motion

**Figure 6:**
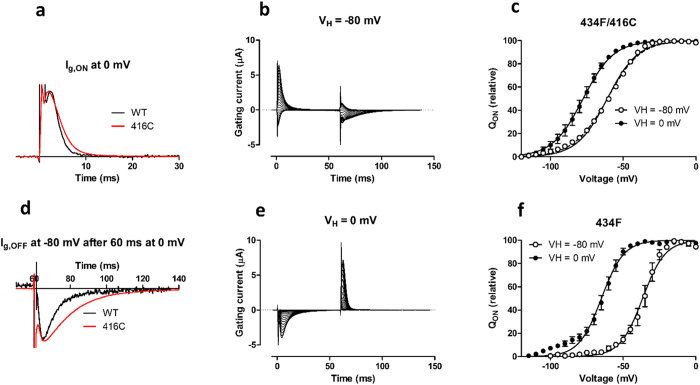
F416C in the pore domain affects molecular motions within the VSD. (**a**) ON gating currents at 0 mV. Holding voltage V_H_ = −80 mV. (**b**) Gating currents of F416C/W434F. V_H_ = −80 mV. (**c**) Charge vs voltage of F416C/W434F calculated from ON gating currents. V_H_ = 0 mV (black symbols), V_H_ = −80 (white symbols). The continuous curves are best fits to Eq. 2 with a shared slope value. *V*_½_(−80 mV) = −61.0 mV, *V*_½_(0 mV) = −77.5 mV. The dashed curve is the best fit of a sum of two Boltzmanns’ curves (Eq. 2), where V_½_ for one component was fixed to −77.5 mV. *V*_½_ for the other component was determined to −58.3 mV. (**d**) OFF gating currents at −80 mV after 60 ms at 0 mV. (**e**) Gating currents of F416C/W434F. V_H_ = 0 mV. (**f**) Charge vs voltage of W434F calculated from ON gating currents. V_H_ = 0 mV (black symbols), V_H_ = −80 mV (white symbols). The continuous curves are best fits to Eq. 2 with a shared slope value. *V*_½_(−80 mV) = −36.6 mV; V_½_(0 mV) = −64.9. All the recordings were done at pH 7.4.

Above, we have shown that alterations in the VSD affect C-type inactivation, and that residues located far away from each other act in a concerted fashion. A critical question is if this concerted action in the pore domain reaches the VSD. To explore this we measured gating currents^27^,^28^, that is intramolecular charge movement within the VSD. Mutations in residue 416 were combined with W434F (to make the pore non-conducting^43^). F416D/W434F did not generate any measurable gating currents. But F416C/W434F generated large gating currents (Fig. 6). The F416C mutation only had a minor effect on the ON gating current measured at 0 mV (Fig. 6a); after the slowly rising phase^22^ the gating current decayed almost exponentially (1.7 ± 0.2 ms, *n* = 4 vs. 2.8 ± 0.8 ms, *n* = 3). The OFF gating current was slower in F416C/W434F compared to W434F (Fig. 6d). After the slowly rising phase (in absolute terms)^22^, the gating current decayed (in absolute terms) almost exponentially (5.2 ± 0.5 ms, *n* = 4 vs. 18.8 ± 4.1 ms, *n* = 3). This suggests a modest stabilization of S4 in the up state by the F416C mutation.

Gating charge movements of VSDs depend on the holding voltage^13^,^15^,^16^,^30^,^44^. The alteration of the gating-charge movement depending on the holding voltage is referred to as a *Q*(*V*) shift. The molecular mechanism is not known but it has been linked to C-type inactivation^13^. However, it is also known to occur in isolated VSDs^30^, which rules out inactivation as a mandatory event. The size of the *Q*(*V*) shift varies from −25 to −70 mV depending on the channel studied and surrounding solutions. Because F416C affected C-type inactivation, we investigated if this mutation also affected the *Q*(*V*) shift in F416C/W434F. The integrated ON and OFF gating currents (i.e. the gating charge) were about equal and the total gating charge was independent of the holding voltage (Fig. 6b,e). The midpoint of the *Q*(*V*) curve was shifted from −61.0 ± 0.3 mV (*n* = 3) to −77.5 ± 0.3 mV (*n* = 3) when the holding voltage was altered from −80 to 0 mV (Fig. 6c). The *Q*(*V*) shift was only −16.5 mV, thus clearly smaller than for wt, −28.3 mV (W434F; *V*_½_ = −36.6 ± 0.5 from a holding voltage of −80 mV; V_½_ = −64.9 ± 0.5 from a holding voltage of 0 mV; *n* = 4) (Fig. 6f). To properly measure the *Q*(*V*) shift, the holding voltage should be from voltages where the gating charges have not started to move. In Fig. 6c it is clear that 16% of the charges have moved at the holding voltage. We tried to keep the holding voltage at more negative values but the cells did not survive long enough to allow stable gating current measurements. To compensate for this less optimal holding voltage, we instead refitted the *Q*(*V*) curves with a sum of two Boltzmann curves^13^ where the midpoint of the second component was fixed to the value for the *Q*(*V*) curve from a holding voltage of 0 mV. The best fit yielded a second component representing 15% of the total gating charge (dashed line in Fig. 6c). Simultaneously V_½_ for the first component was shifted from −61.0 to −58.3 mV. Thus, the corrected *Q*(*V*) shift should be −19.2 instead of −16.5 mV, but still significantly smaller than for WT. Thus, a mutation in the pore domain at the extracellular end of S5 modifies gating charge movements and the size of the *Q*(*V*) shift.

## Discussion

The present investigation, focused on the VSD-to-pore domain connection, suggests the following:

1. A centrifugal motion of 416 (meaning to flee from the center) is associated with selectivity filter widening, K^+^ loss, and channel inactivation. Plenty of data support this: (i) A short negatively charged residue at 416, probably attracted to R365, accelerated C-type inactivation. (ii) A Cd^2+^ bridge between F416C and R365C accelerated C-type inactivation. (iii) A rotation and/or tilt of S4 altered the rate of C-type inactivation. (iv) A centrifugal pulling of residue 416 in molecular dynamics simulations widened the selectivity filter and altered the coordination of bound K^+^ ions. (v) A restoration of the filter structure was hampered by a Cd^2+^ bridge inside the VSD.
2. Molecular motions in the extracellular portion of the channel associated with C-type inactivation occur in a concerted fashion. This suggests that a pore widening leads to centrifugal motion of 416 and conversely, a centrifugal motion of 416 leads to a filter widening.
3. An alteration in residue 416 in the pore domain modifies the *Q*(*V*) shift of the VSD.

**Figure 7:**
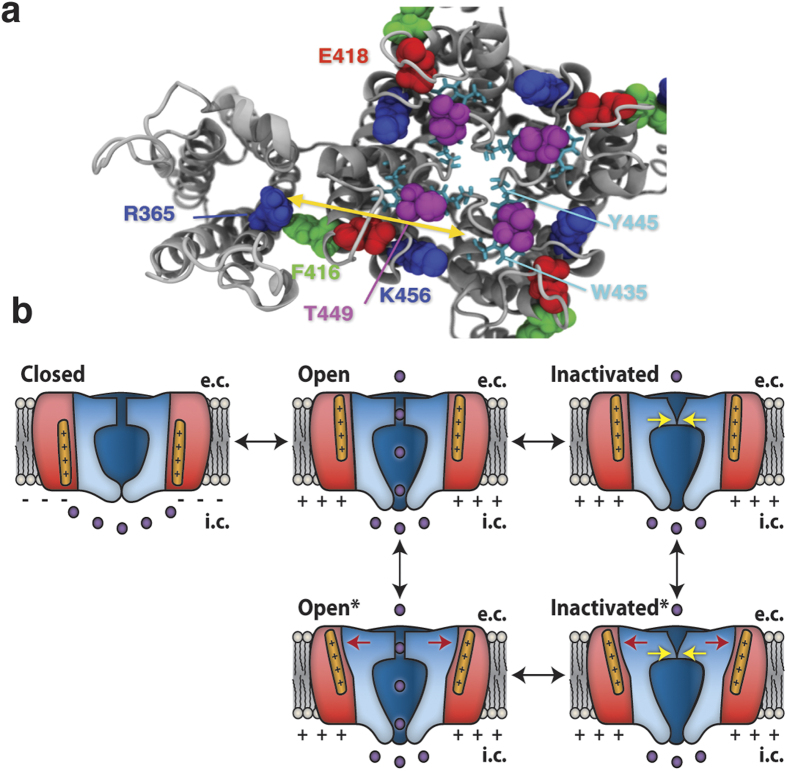
Mechanisms for C-type inactivation. (**a**) Molecular structure of one VSD (left) and the pore domain (right). Important residues explored in this study are space filled. Yellow double-directed arrow denotes approximate path for the molecular signal. (**b**) Postulated inactivation and *Q*(*V*)-shift pathways. Pore domain (blue), VSD (red), S4 (orange). Depolarization shifts the channel from a *closed* state to an *open* state. Prolonged depolarization either (1) rearranges the selectivity filter (*inactivated* state), and then alters the outer pore domain and S4 (inactivated* state), or (2) alters the outer pore domain and S4 (open* state), and then rearranges of the selectivity filter (inactivated* state).

We propose the following scenario: The outward movement of S4 rotates S6 and opens the intracellular gate^45^,^46^. The S6 rotation triggers a concerted process where the filter widening, the K^+^ loss, the pore-to-S6 loop rotation^15^, and the centrifugal motion of residue 416 are closely coupled (Fig. 7a). The centrifugal motion of residue 416 stabilize S4 in an up state and consequently shift the *Q*(*V*) curve in negative direction along the voltage axis during slow inactivation.

The *Q*(*V*) curve in many voltage-gated ion channels and isolated VSDs (i.e. without a pore domain) depends on the holding voltage^13^,^15^,^16^,^30^,^44^. In other words, the gating-charge movement of the VSD depends on the voltage prehistory. The time course of the *Q*(*V*) shift, roughly follows the time course C-type inactivation in some channels^13^ but the link is not obligatory (see below). Thus, the *Q*(*V*) shift is probably caused by an alteration in the molecular structure of the VSD occurring during C-type inactivation. However, the *Q*(*V*) shift is also found in VSDs lacking the pore domain^30^ (which per definition cannot inactivate), and in channels where C-type inactivation is prevented^16^,^47^. Conversely, in channels lacking a VSD, a C-type like inactivation is present in the selectivity filter^11^. Thus, the *Q*(*V*) shift and C-type inactivation are closely linked in some ion channels but the two molecular events can also occur independent of each other.

Figure 7b illustrates the relation between the *Q*(*V*) shift and C-type inactivation. Activation (outward movement of S4; *Closed* → *Open*) typically takes a few milliseconds, while the *Q*(*V*) shift (vertical transitions) and C-type inactivation (horizontal transitions) takes a few seconds. Either there is a tight link between the *Q*(*V*) shift and the C-type inactivation, or the two processes can occur relatively independent of each other. If there is a tight link, the *Inactivated* and Open* states are only briefly occupied. Experimental data on the time course of the inactivation and the *Q*(*V*) shift^13^ suggests they are tightly linked, and this is supported by our data that residues covering a large part of the channel, from the selectivity filter to the VSD, act in a concerted fashion. On the other hand, the W434F mutation, close to the selectivity filter, accelerates the inactivation by a factor of >10^6^ 48, but does not eliminate the *Q*(*V*) shift^13^, suggesting that the *Inactivated* state (Fig. 7b) must be semi-stable. Thus, we propose that there is a wide range of VSD-to pore couplings, where Shaker wt has a relatively tight coupling. In Shaker, we suggest that a structural swelling of the pore domain is directly linked to a widening of the selectivity filter and the *Q*(*V*) shift, and thus that the *Inactivated* and Open* states are suggested to be only briefly occupied (Fig. 7b).

The link between the VSD and the pore domain with respect to C-type inactivation and the *Q*(*V*) shift is important in normal physiology and in pathology. A mutation in S1 in the Kv1.1 channel gives rise to episodic ataxia and the molecular disease mechanism has, in part, been shown to affect C-type inactivation^49^. Pace-maker HCN channels, which are responsible for triggering action potentials in the sinoatrial node of the heart, undergo a prominent *Q*(*V*) shift (or mode shift) during each heart cycle; a reduced mode shift is suggested to cause cardiac arrhythmia^16^,^50^. While the HCN channel is a distant relative to the voltage-gated K channels, it has been suggested that the mechanism of C-type inactivation and the mode shift share similar features^16^. Thus, elucidation of the molecular mechanism underlying C-type inactivation could suggest new treatments for channel-related diseases.

In conclusion, the present study suggests a direct linkage between the VSD and the pore domain, which affects C-type inactivation. Both experimental and modelling data suggests that centrifugal pulling in the pore domain leads to C-type inactivation, and that the resulting structural alteration in the pore domain affects the VSD function. This linkage is suggested to play a role in diseases and therefore also in its treatments.

## Material and Methods

### Molecular biology

All animal experiments were approved by Linköping’s Animal Care and Use Committee and followed international guidelines. Experiments were carried out on the Shaker H4 channel (accession number NM_167595.3)^51^ made incapable of fast inactivation by the Δ(6–46) deletion^52^ that was inserted into the Bluescript II KS(+) plasmid. This channel is referred to Shaker wt in this study. Point mutations were introduced using QuikChange Site-Directed Mutagenesis kit (Agilent Technologies) and verified by sequencing. cRNA was synthesised from DNA using the mMessage mMachine T7 kit (Ambion, Austin, TX).

### Preparation and injection of oocytes

African clawed frogs (*Xenopus laevis*) were anesthetized with 1.4 g/L ethyl 3-aminobenzoate methanesulfonate salt (tricaine). After an incision through the abdomen a batch of oocytes were removed and the wound stitched together. Clusters of oocytes were separated by incubation for ~1 h in a Ca^2+^-free OR-2 solution (in mM: 82.5 NaCl, 2 KCl, 5 HEPES, and 1 MgCl_2_; pH adjusted to 7.4 by NaOH) containing Liberase Blendzyme. The oocytes were then incubated at 8 °C either in modified Barth’s solution (MBS; in mM: 88 NaCl, 1 KCl, 2.4 NaHCO_3_, 15 HEPES, 0.33 Ca(NO_3_)_2_, 0.41 CaCl_2_, and 0.82 MgSO_4_; pH adjusted to 7.6 by NaOH) supplemented with penicillin (25 U/ml), streptomycin (25 μg/ml), and sodium pyruvate (2.5 mM) or in SBS (in mM: 88 NaCl, 1 KCl, 0.4 CaCl_2_, 0.33 Ca(NO_3_)_2_, 0.8 MgSO_4_, 5 Tris-Hcl, and 2.4 NaHCO_3_,) 2–24 hours before injection. 50 nl of cRNA was injected into each oocyte using a Nanoject injector (Drummond Scientific, Broomall, PA). Injected oocytes were kept at 8 °C in MBS or SBS. All chemicals were supplied from Sigma-Aldrich (Stockholm, Sweden) if not stated otherwise. For double cysteine mutants, 0.5 mM DTT was added to the MBS or SBS to prevent disulfide-bond formation during incubation. Separated oocytes were ordered from ecocyte (Castrop-Rauxel, Germany). Ordered oocytes were injected and stored as described above. Results from experiments were independent of storage solution and oocyte origin.

### Electrophysiology

All manual electrophysiological recordings were performed at room temperature (20–23 °C), using a CA-1B amplifier (Dagan Corporation) and a Digidata 1322A or 1440A digitizer and pClamp 10 software (Molecular Devices, Inc., Sunnyvale, CA, USA). The amplifier’s leak and capacitance compensation were used, and the currents were low-pass filtered at 5 kHz. The oocyte was placed in a bath surrounded by 1K extracellular solution that contained (in mM): 88 NaCl, 1 KCl, 15 HEPES, 0.4 CaCl_2_, and 0.8 MgCl_2_, pH adjusted to 7.4 by NaOH (reaching a Na^+^ concentration of ~100 mM). Control solution was added to the bath using a gravity driven perfusion system. Solutions of 10 μM CdCl_2_, 70 μM DHA, and 70 μM DHA-me were prepared in control solution (unless stated otherwise). To study channel voltage dependence and kinetics, steady-state currents were achieved by stepping to voltages typically between −80 mV and +100 mV in 10 mV increments with 100 ms long pulses. To study channel C-type inactivation, currents were recorder by stepping to voltages between −40 mV and +80 mV in 20 mV increments with 10 s long pulses. To study gating currents the membrane voltage was stepped to voltages between −150 mV and +100 mV for 60 ms from a holding voltage of either −80 mV or 0 mV. The membrane capacitance was compensated at 0 mV.

### Analysis of electrophysiological data

The electrophysiological data were processed using Clampfit 10.4 (Molecular Devices, LLC.) and GraphPad Prism 5 (GraphPad Software, inc) software. The conductance *G*_K_(*V*) was calculated as


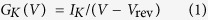


where *I*_K_ is the average current from the steady-state phase at the end of each 100-ms pulse, *V* is the absolute membrane voltage, and *V*_rev_ is the reversal potential for K^+^ (set to −80 mV for the oocytes). These data were fitted to a Boltzmann equation


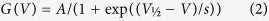


where *A* is amplitude of the curve, *V*_½_ is the midpoint voltage, and *s* is the slope.

To calculate the Cd^2+^-induced effect on the inactivation transitions we assumed the following state diagram:


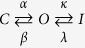


The rate constants α and β are fast (ms time scale), while λ and κ are slow (s time scale). If the open probability is high, i.e. α » β, then λ and κ can be calculated. The decaying phase of the K current (I_K_) is fitted to


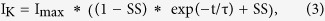


where I_max_ is the current at the start of the fit (close to peak current), SS is the expected steady-state current, and τ is the time constant of the inactivation. τ=1 / (κ + λ), and SS=λ/(κ + λ), and thus, the rate constants can be calculated:


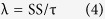



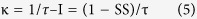


*κ* and λ where calculated from current traces at very positive voltages to ensure high open probability even in the mutant with the most right shifted voltage dependence (Supplementary Table I). However, for some mutants, Cd^2+^ increased the maximum current slightly, thereby altering the time constant of inactivation. Thus, the calculations were modified for every experiment to take into account the increase in current.

To determine if two non-interacting residues in a subunit act in concert to promote C-type inactivation we studied the rate of inactivation of combinations of the mutants shown to affect C-type inactivation (see Supplementary Information for more details). If the two residues (A and B) work in concert we expect that


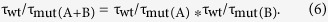


If the two non-interacting residues instead affect C-type inactivation independent of each other, the mutation causing the fastest C-type inactivation will dominate the process. Thus, if τ_A_ < τ_B_, then


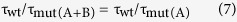


To analyze gating currents, a leakage current correction was performed off line. The gating charge was calculated from 1.5 ms after onset of pulse (to exclude the majority of the capacitive current) to the end of the ON pulse. *Q*(*V*) was normalized and plotted as a percentage of the maximum. Data are means ± SEM. The effect of specific mutations on the time constant and steady state level of currents was compared to WT values using one-way ANOVA (Dunnett’s multiple comparison test). P < 0.05 is considered statistically significant.

### Molecular modeling and simulation

An homology model of Shaker was constructed based on the X-ray structure of the Kv1.2–2.1 chimera (PDB entry 2R9R) in ROSETTA. The model was embedded in a POPC bilayer with explicit solvent using the CHARMM36 force field for both protein and lipids^53^. The simulations where carried out using GROMACS 5.0.3^54^, using particle-mesh Ewald electrostatics and a 1 nm cut-off for both electrostatics and van der Waals interactions. A 2fs time step was used, with all bond lengths constrained using the LINCS algorithm. Temperature was kept at 300K using the Bussi v-rescale thermostat, and semi isotropic pressure coupling applied with the Parrinello-Rahman barostat. All systems were subject to steepest descent energy minimization and then relaxed with 1000 kJ/mol/nm^2^ position restraints on heavy atoms for 50 ns prior to starting production simulations.

## Additional Information

**How to cite this article**: Conti, L. *et al*. Reciprocal voltage sensor-to-pore coupling leads to potassium channel C-type inactivation. *Sci. Rep.* **6**, 27562; doi: 10.1038/srep27562 (2016).

## Supplementary Material

Supplementary Information

## References

[b1] Lin W.-H. & Baines R. A. Regulation of membrane excitability: a convergence on voltage-gated sodium conductance. Mol. Neurobiol. 51, 57–67 (2015). 24677068 10.1007/s12035-014-8674-0 PMC4309913

[b2] Stein M. . Combined reduction of intercellular coupling and membrane excitability differentially affects transverse and longitudinal cardiac conduction. Cardiovasc. Res. 83, 52–60 (2009). 19389723 10.1093/cvr/cvp124

[b3] Sanguinetti M. C. & Spector P. S. Potassium channelopathies. Neuropharmacology 36, 755–762 (1997). 9225302 10.1016/s0028-3908(97)00029-4

[b4] Kurata H. T. & Fedida D. A structural interpretation of voltage-gated potassium channel inactivation. Prog. Biophys. Mol. Biol. 92, 185–208 (2006). 16316679 10.1016/j.pbiomolbio.2005.10.001

[b5] Liu Y., Jurman M. E. & Yellen G. Dynamic rearrangement of the outer mouth of a K^+^ channel during gating. Neuron 16, 859–867 (1996). 8608004 10.1016/s0896-6273(00)80106-3

[b6] Starkus J. G., Kuschel L., Rayner M. D. & Heinemann S. H. Ion conduction through C-type inactivated Shaker channels. J. Gen. Physiol. 110, 539–550 (1997). 9348326 10.1085/jgp.110.5.539 PMC2229384

[b7] López-Barneo J., Hoshi T., Heinemann S. H. & Aldrich R. W. Effects of external cations and mutations in the pore region on C-type inactivation of Shaker potassium channels. Receptors Channels 1, 61–71 (1993). 8081712

[b8] Hoshi T., Zagotta W. N. & Aldrich R. W. Two types of inactivation in Shaker K^+^ channels: effects of alterations in the carboxy-terminal region. Neuron 7, 547–556 (1991). 1931050 10.1016/0896-6273(91)90367-9

[b9] Ogielska E. M. . Cooperative subunit interactions in C-type inactivation of K channels. Biophys. J. 69, 2449–2457 (1995). 8599651 10.1016/S0006-3495(95)80114-1 PMC1236482

[b10] Immke D., Kiss L., LoTurco J. & Korn S. J. Influence of non-P region domains on selectivity filter properties in voltage-gated K^+^ channels. Receptors Channels 6, 179–188 (1998). 10100326

[b11] Cuello L. G., Jogini V., Cortes D. M. & Perozo E. Structural mechanism of C-type inactivation in K(+) channels. Nature 466, 203–208 (2010). 20613835 10.1038/nature09153 PMC3033749

[b12] Cordero-Morales J. F. . Molecular determinants of gating at the potassium-channel selectivity filter. Nat. Struct. Mol. Biol. 13, 311–318 (2006). 16532009 10.1038/nsmb1069

[b13] Olcese R., Latorre R., Toro L., Bezanilla F. & Stefani E. Correlation between Charge Movement and Ionic Current during Slow Inactivation in Shaker K^+^ Channels. J. Gen. Physiol. 110, 579–589 (1997). 9348329 10.1085/jgp.110.5.579 PMC2229383

[b14] Loots E. & Isacoff E. Y. Protein rearrangements underlying slow inactivation of the Shaker K^+^ channel. J. Gen. Physiol. 112, 377–389 (1998). 9758858 10.1085/jgp.112.4.377 PMC2229423

[b15] Larsson H. P. & Elinder F. A conserved glutamate is important for slow inactivation in K^+^ channels. Neuron 27, 573–583 (2000). 11055439 10.1016/s0896-6273(00)00067-2

[b16] Männikkö R., Pandey S., Larsson H. P. & Elinder F. Hysteresis in the voltage dependence of HCN channels: conversion between two modes affects pacemaker properties. J. Gen. Physiol. 125, 305–326 (2005). 15710913 10.1085/jgp.200409130 PMC2234019

[b17] Loboda A., Melishchuk A. & Armstrong C. Dilated and defunct K channels in the absence of K^+^. Biophys. J. 80, 2704–2714 (2001). 11371446 10.1016/S0006-3495(01)76239-X PMC1301457

[b18] Köpfer D. A. . Ion permeation in K^+^ channels occurs by direct Coulomb knock-on. Science 346, 352–355 (2014). 25324389 10.1126/science.1254840

[b19] Ostmeyer J., Chakrapani S., Pan A. C., Perozo E. & Roux B. Recovery from slow inactivation in K^+^ channels is controlled by water molecules. Nature 501, 121–124 (2013). 23892782 10.1038/nature12395 PMC3799803

[b20] Hoshi T. & Armstrong C. M. C-type inactivation of voltage-gated K^+^ channels: pore constriction or dilation? J. Gen. Physiol. 141, 151–160 (2013). 23319730 10.1085/jgp.201210888 PMC3557304

[b21] Zhou Y., Morais-Cabral J. H., Kaufman A. & MacKinnon R. Chemistry of ion coordination and hydration revealed by a K^+^ channel-Fab complex at 2.0 A resolution. Nature 414, 43–48 (2001). 11689936 10.1038/35102009

[b22] Bezanilla F., Perozo E., Papazian D. M. & Stefani E. Molecular basis of gating charge immobilization in Shaker potassium channels. Science 254, 679–683 (1991). 1948047 10.1126/science.1948047

[b23] Larsson H. P., Baker O. S., Dhillon D. S. & Isacoff E. Y. Transmembrane Movement of the Shaker K^+^ Channel S4. Neuron 16, 387–397 (1996). 8789953 10.1016/s0896-6273(00)80056-2

[b24] Long S. B., Tao X., Campbell E. B. & MacKinnon R. Atomic structure of a voltage-dependent K^+^ channel in a lipid membrane-like environment. Nature 450, 376–382 (2007). 18004376 10.1038/nature06265

[b25] Henrion U. . Tracking a complete voltage-sensor cycle with metal-ion bridges. Proc. Natl. Acad. Sci. USA 109, 8552–8557 (2012). 22538811 10.1073/pnas.1116938109 PMC3365220

[b26] Vargas E. . An emerging consensus on voltage-dependent gating from computational modeling and molecular dynamics simulations. J. Gen. Physiol. 140, 587–594 (2012). 23183694 10.1085/jgp.201210873 PMC3514734

[b27] Armstrong C. M. & Bezanilla F. Charge movement associated with the opening and closing of the activation gates of the Na channels. J. Gen. Physiol. 63, 533–552 (1974). 4824995 10.1085/jgp.63.5.533 PMC2203568

[b28] Keynes R. D. & Rojas E. Kinetics and steady-state properties of the charged system controlling sodium conductance in the squid giant axon. J. Physiol. 239, 393–434 (1974). 4414038 10.1113/jphysiol.1974.sp010575 PMC1330930

[b29] Bezanilla F., White M. M. & Taylor R. E. Gating currents associated with potassium channel activation. Nature 296, 657–659 (1982). 6280059 10.1038/296657a0

[b30] Villalba-Galea C. A., Sandtner W., Starace D. M. & Bezanilla F. S4-based voltage sensors have three major conformations. Proc. Natl. Acad. Sci. USA 105, 17600–17607 (2008). 18818307 10.1073/pnas.0807387105 PMC2584729

[b31] Ortega-Sáenz P., Pardal R., Castellano A. & López-Barneo J. Collapse of conductance is prevented by a glutamate residue conserved in voltage-dependent K(+) channels. J. Gen. Physiol. 116, 181–190 (2000). 10919865 10.1085/jgp.116.2.181 PMC2229493

[b32] Armstrong C. M. & Hoshi T. K^+^ channel gating: C-type inactivation is enhanced by calcium or lanthanum outside. J. Gen. Physiol. 144, 221–230 (2014). 25156116 10.1085/jgp.201411223 PMC4144669

[b33] Lee S.-Y., Banerjee A. & MacKinnon R. Two separate interfaces between the voltage sensor and pore are required for the function of voltage-dependent K(+) channels. PLos Biol. 7, e47 (2009). 19260762 10.1371/journal.pbio.1000047 PMC2650729

[b34] Broomand A., Männikkö R., Larsson H. P. & Elinder F. Molecular movement of the voltage sensor in a K channel. J. Gen. Physiol. 122, 741–748 (2003). 14610021 10.1085/jgp.200308927 PMC2229587

[b35] Shem-Ad T., Irit O. & Yifrach O. Inter-subunit interactions across the upper voltage sensing-pore domain interface contribute to the concerted pore opening transition of Kv channels. PLos One 8, e82253 (2013). 24340010 10.1371/journal.pone.0082253 PMC3858418

[b36] Mckeown L., Burnham M. P., Hodson C. & Jones O. T. Identification of an evolutionarily conserved extracellular threonine residue critical for surface expression and its potential coupling of adjacent voltage-sensing and gating domains in voltage-gated potassium channels. J. Biol. Chem. 283, 30421–30432 (2008). 18640987 10.1074/jbc.M708921200 PMC2662089

[b37] Füll Y., Seebohm G., Lerche H. & Maljevic S. A conserved threonine in the S1-S2 loop of KV7.2 and K V7.3 channels regulates voltage-dependent activation. Pflüg. Arch. Eur. J. Physiol. 465, 797–804 (2013). 10.1007/s00424-012-1184-x 23271449

[b38] Börjesson S. I. & Elinder F. An electrostatic potassium channel opener targeting the final voltage sensor transition. J. Gen. Physiol. 137, 563–577 (2011). 21624947 10.1085/jgp.201110599 PMC3105513

[b39] Tombola F., Pathak M. M., Gorostiza P. & Isacoff E. Y. The twisted ion-permeation pathway of a resting voltage-sensing domain. Nature 445, 546–549 (2007). 17187057 10.1038/nature05396

[b40] Lin M. A., Hsieh J.-Y., Mock A. F. & Papazian D. M. R. 1. in the Shaker S4 occupies the gating charge transfer center in the resting state. J. Gen. Physiol. 138, 155–163 (2011). 21788609 10.1085/jgp.201110642 PMC3149438

[b41] Ottosson N. E., Liin S. I. & Elinder F. Drug-induced ion channel opening tuned by the voltage sensor charge profile. J. Gen. Physiol. 143, 173–182 (2014). 24420769 10.1085/jgp.201311087 PMC4001773

[b42] Liin S. I. . Polyunsaturated fatty acid analogs act antiarrhythmically on the cardiac IKs channel. Proc. Natl. Acad. Sci. USA 112, 5714–5719 (2015). 25901329 10.1073/pnas.1503488112 PMC4426425

[b43] Perozo E., MacKinnon R., Bezanilla F. & Stefani E. Gating currents from a nonconducting mutant reveal open-closed conformations in Shaker K^+^ channels. Neuron 11, 353–358 (1993). 8352943 10.1016/0896-6273(93)90190-3

[b44] Bezanilla F., Taylor R. E. & Fernández J. M. Distribution and kinetics of membrane dielectric polarization. 1. Long-term inactivation of gating currents. J. Gen. Physiol. 79, 21–40 (1982). 7061986 10.1085/jgp.79.1.21 PMC2215491

[b45] Elinder F., Männikkö R. & Larsson H. P. S4 charges move close to residues in the pore domain during activation in a K channel. J. Gen. Physiol. 118, 1–10 (2001). 11429439 10.1085/jgp.118.1.1 PMC2233763

[b46] Long S. B., Campbell E. B. & Mackinnon R. Voltage sensor of Kv1.2: structural basis of electromechanical coupling. Science 309, 903–908 (2005). 16002579 10.1126/science.1116270

[b47] Olcese R., Sigg D., Latorre R., Bezanilla F. & Stefani E. A conducting state with properties of a slow inactivated state in a shaker K(+) channel mutant. J. Gen. Physiol. 117, 149–163 (2001). 11158167 10.1085/jgp.117.2.149 PMC2217242

[b48] Yang Y., Yan Y. & Sigworth F. J. How does the W434F mutation block current in Shaker potassium channels? J. Gen. Physiol. 109, 779–789 (1997). 9222903 10.1085/jgp.109.6.779 PMC2217041

[b49] Petitjean D., Kalstrup T., Zhao J. & Blunck R. A Disease Mutation Causing Episodic Ataxia Type I in the S1 Links Directly to the Voltage Sensor and the Selectivity Filter in Kv Channels. J. Neurosci. Off. J. Soc. Neurosci. 35, 12198–12206 (2015). 10.1523/JNEUROSCI.1419-15.2015 PMC6605306 26338330

[b50] Elinder F., Männikkö R., Pandey S. & Larsson H. P. Mode shifts in the voltage gating of the mouse and human HCN2 and HCN4 channels. J. Physiol. 575, 417–431 (2006). 16777944 10.1113/jphysiol.2006.110437 PMC1819464

[b51] Kamb A., Iverson L. E. & Tanouye M. A. Molecular characterization of Shaker, a Drosophila gene that encodes a potassium channel. Cell 50, 405–413 (1987). 2440582 10.1016/0092-8674(87)90494-6

[b52] Hoshi T., Zagotta W. N. & Aldrich R. W. Biophysical and molecular mechanisms of Shaker potassium channel inactivation. Science 250, 533–538 (1990). 2122519 10.1126/science.2122519

[b53] Best R. B. . Optimization of the additive CHARMM all-atom protein force field targeting improved sampling of the backbone ϕ, ψ and side-chain χ(1) and χ(2) dihedral angles. J. Chem. Theory Comput. 8, 3257–3273 (2012). 23341755 10.1021/ct300400x PMC3549273

[b54] Abraham M. J. . GROMACS: High performance molecular simulations through multi-level parallelism from laptops to supercomputers. SoftwareX 1–2, 19–25 (2015).

