# Supplementary material for: Reciprocal voltage sensor-to-pore coupling leads to potassium channel C-type inactivation
Source: Sci Rep. 2016 Jun 9;6:27562. doi: 10.1038/srep27562 (PMC4899724; doi:10.1038/srep27562)
Supplement: Supplementary Information [file srep27562-s1.pdf]

Reciprocal voltage sensor-to-pore coupling leads to potassium channel C-type inactivation

Luca Conti[1], Jakob Renhorn[1], Anders Gabrielsson[2], Fredrik Turesson[1], Sara I Liin[1], Erik Lindahl[2,3] & Fredrik Elinder[1]

[1]Department of Clinical and Experimental Medicine, Linköping University, Linköping, Sweden
[2]Theoretical and Computational Biophysics, Department of Theoretical Physics, KTH Royal Institute of Technology, Stockholm, Sweden
[3]Science for Life Laboratory, Department of Biochemistry and Biophysics, Stockholm University, Stockholm, Sweden

SUPPLEMENTARY TEXT

Concerted vs. non-concerted inactivation

To test if a single conformational alteration of the channel protein involves a larger region of the protein or not, we performed a mutational analysis. In general, if the effects are global (involving a larger region), the alteration in energy of the single mutations can be summed together to predict the effect of the double mutation. If the effect are local, the effects of the mutations act independent of each other, and the energies cannot be added together.

Energetics of single mutations

The alteration in kinetics caused by a single mutation depends on the energy alteration introduced by a mutation. For instance a mutation 1 can strengthen or weaken of a chemical bond that has to be broken for the channel to go from the open state to the inactivated state. The alteration in kinetics is described by

$$k_{wt}/k_{mut1} = A * \exp(-E_{mut1}/k_B), \qquad (S1)$$

where k_{wt} is the inactivation rate in the wild-type channel wt, and where k_{mut1} is the inactivation rate in mutant 1. A is the attempt frequency (10^{12} s^{-1}), E_{mut1} is the difference in energy between wt and mutant 1 with respect to bond 1, and k_B is Boltzmann's constant.

Energetics of two mutations acting in concert

If two mutations are introduced in the protein and if both has to be broken more or less simultaneously to inactivate the channel, the two energies should be added:

$$k_{wt}/k_{mut1+mut2} = A * \exp(-(E_{mut1}+E_{mut2})/k_B) =$$
$$= A * \exp(-E_{mut1}/k_B) * \exp(-E_{mut2}/k_B) = k_{wt}/k_{mut1} * k_{wt}/k_{mut2} \qquad (S2)$$

For instance, if mutation 1 speeds up C-type inactivation by a factor of 10 and mutation 2 also speeds up the inactivation by a factor of 10, then the double mutation should speed up the inactivation by a factor of 100. If, on the other hand, mutation 2 slows down the inactivation by a factor of 10, then the overall inactivation rate should not be affected.

Energetics of two mutations acting independent of each other

If two or several independent chemical bonds have to be broken to let the channel inactivate, these bonds can be considered to control independent “gates” that close the channel. If one mutation affects a bond to be broken quickly, the addition of another fast mutation is not expected to speed up the overall rate of inactivation very much. A simple calculation can be performed based on the following state diagram

$$I \xleftarrow{k_1} O \xrightarrow{k_2} I$$

There are two paths leading from the open state to the inactivated state(s). k_1 and k_2 are not known but the overall rate inactivation rate k_{wt} is known:

$$k_{wt} = k_1 + k_2. \qquad \text{(S3)}$$

The effect of mutation 1 on the overall inactivation rate can be measured but the exact effect on k_1 is not known but can be called k_1*.

$$k_{mut1} = k_1^* + k_2 \qquad \text{(S4)}$$

The same reasoning holds for mutation 2 and the double mutation 12.

$$k_{mut2} = k_1 + k_2^* \qquad \text{(S5)}$$

$$k_{mut1,mut2} = k_1^* + k_2^* \qquad \text{(S6)}$$

Equations S3-S6 yields

$$k_{mut1,mut2} = k_{mut1} + k_{mut2} - k_{wt} \qquad \text{(S7)}$$

If f_{mutx} denotes the factor that mutation x speeds up C-type inactivation, equation 7 can be rewritten as

$$f_{mut1,mut2} = f_{mut1} + f_{mut2} - 1 \qquad \text{(S8)}$$

For example, if mutation 1 speeds up C-type inactivation by a factor of 10 and mutation 2 also speeds up C-type inactivation by a factor of 10, then the double mutation is expected to speed up the inactivation by a factor of 19, which is far away from a factor of 100 expected from a concerted action. Equation S8 holds for calculations when at least one of the mutation speeds up slow inactivation. If both single mutations slows down the C-type inactivation, more complicated diagrams must be employed.

SUPPLEMENTARY FIGURES

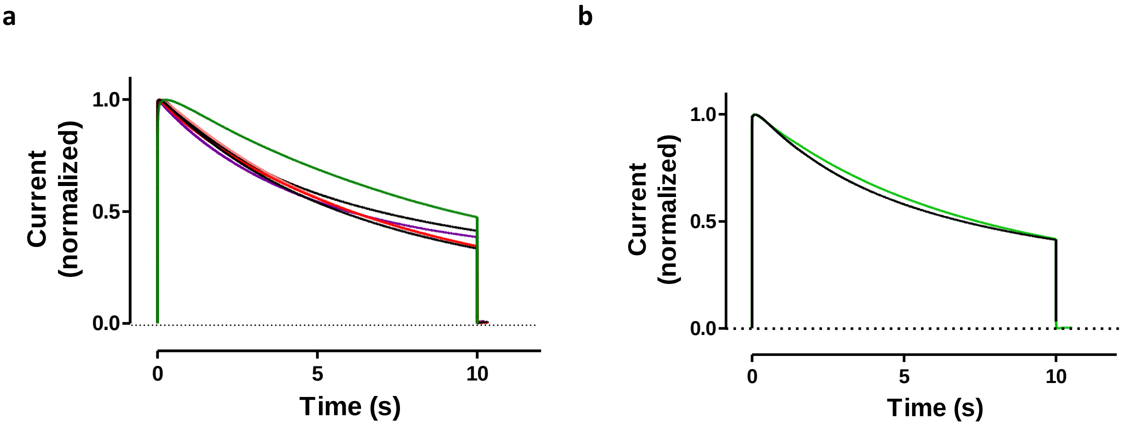

Supplementary figure S1. Residue F416 is involved in the communication between the VSD and the pore domain. **(a)** Superimposed current traces of wild type (dark), F416C (blue), F416L (red), F416Q (green), F416W (pink), F416A (violet) recorded at +80 mV, pH 7.4. **(b)** The mutation F416H (green) recorded at +80 mV, pH 6 does not affect slow inactivation of the channel compared to wild type recorded at pH 6 (dark). Holding potential = –80 mV.

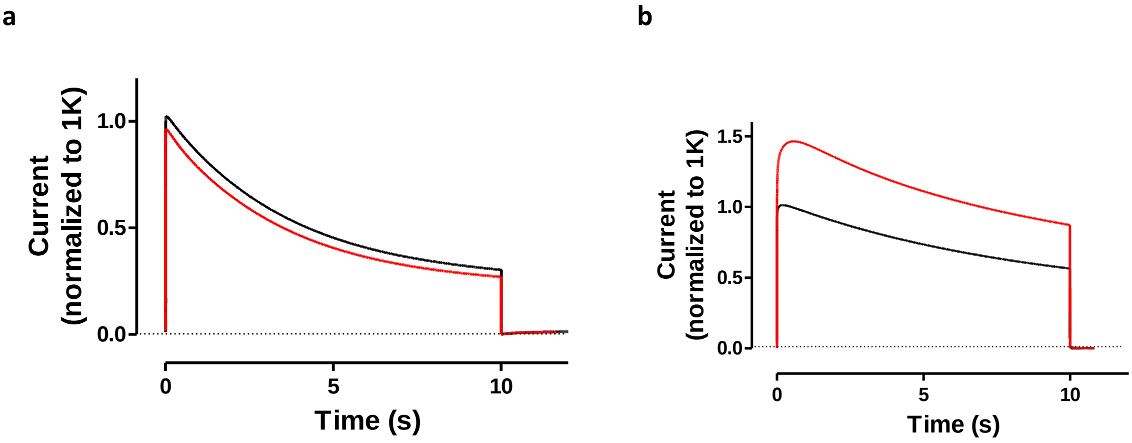

Supplementary figure S2. 10 μM Cd^{2+} (red) do not affect the time course of slow inactivation of 416C **(a)** and 365C **(b)**. Holding potential = –80 mV. Pulse to +80 mV.

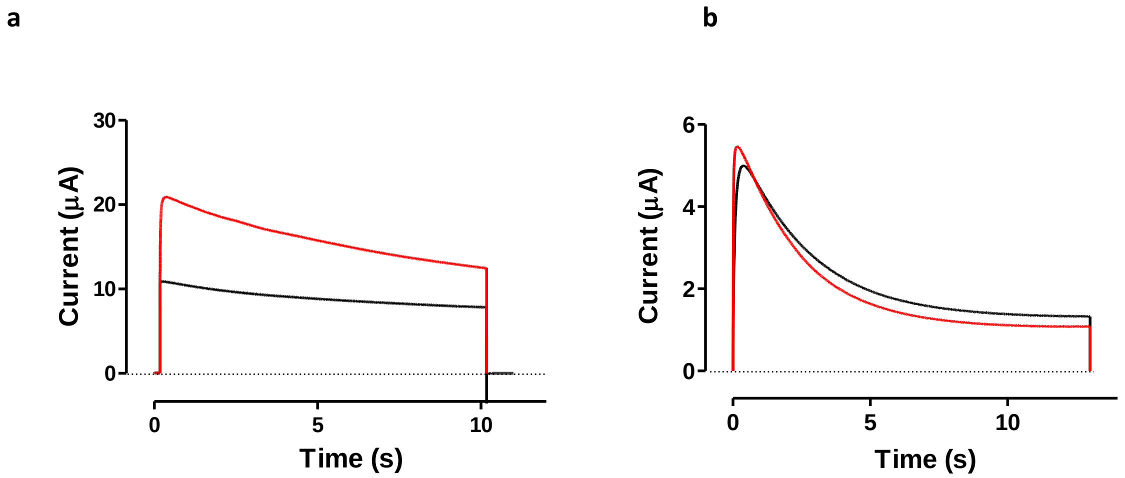

Supplementary figure S3. A Cd^{2+} bridge between 326C/368C **(a)** or 326C/365C **(b)** does not affect C-type inactivation.

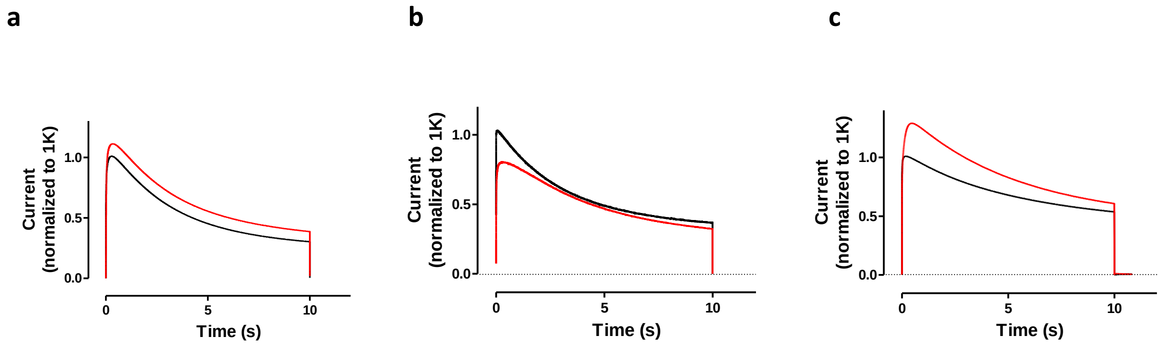

Supplementary figure S4. 10 µM Cd^{2+} (red) applied at –80 mV does not affect the C-type inactivation of single mutants 326C **(a)**, 327C **(b)**, 368C **(c)**. Holding potential = –80 mV. Pulse to +80 mV.

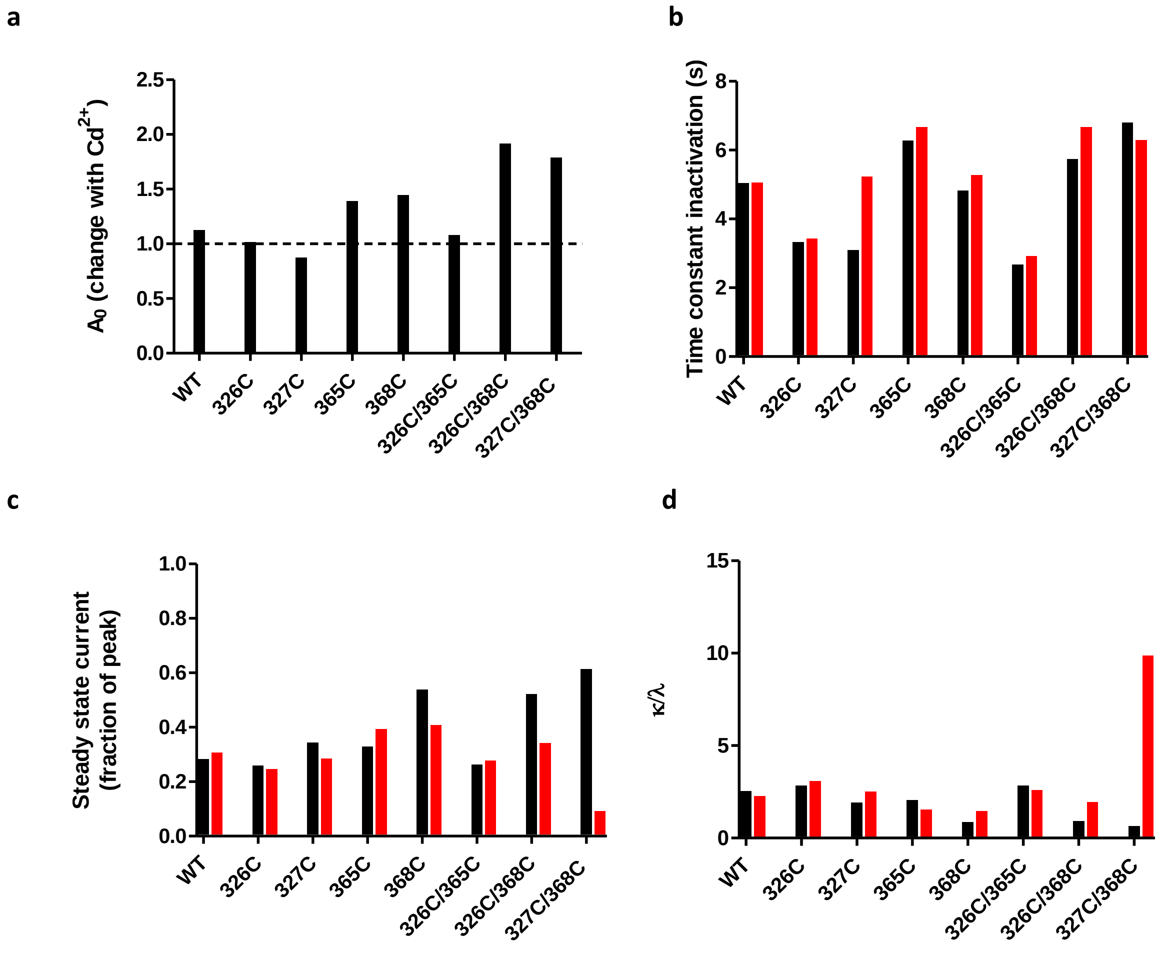

Supplementary figure S5. 10 μM Cd^{2+} applied to the double mutant 327C/368C affects the steady state currents of slow inactivation. **(a)** Change of current amplitude by 10 μM Cd^{2+} in different mutations. **(b)** Values of time constants before (black) and after the application of 10 μM Cd^{2+} (red) for all the mutants examined. **(c)** Values of steady state currents before (black) and after the application of Cd^{2+} (red). (**d**) Values of the ratio between rate constants λ and κ before and after the application of Cd^{2+} (red). The ratio κ/λ is significantly increased in the double mutant 327C/368C after the application of Cd^{2+}. Recordings done with holding potential = –80 mV. Ten s pulse to +80 mV.

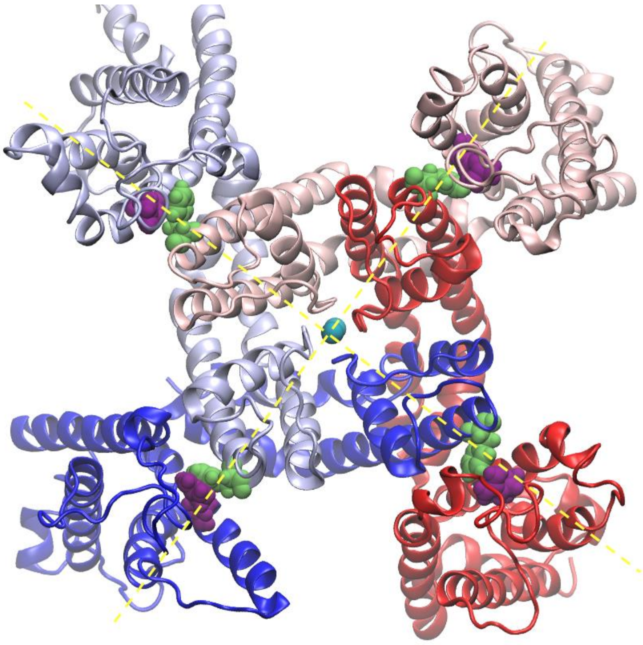

Supplementary figure S6. The pull direction between pairs of F416 (green) roughly coincides with the direction of a Cd^{2+}-bridge between F416 and R362 (purple).

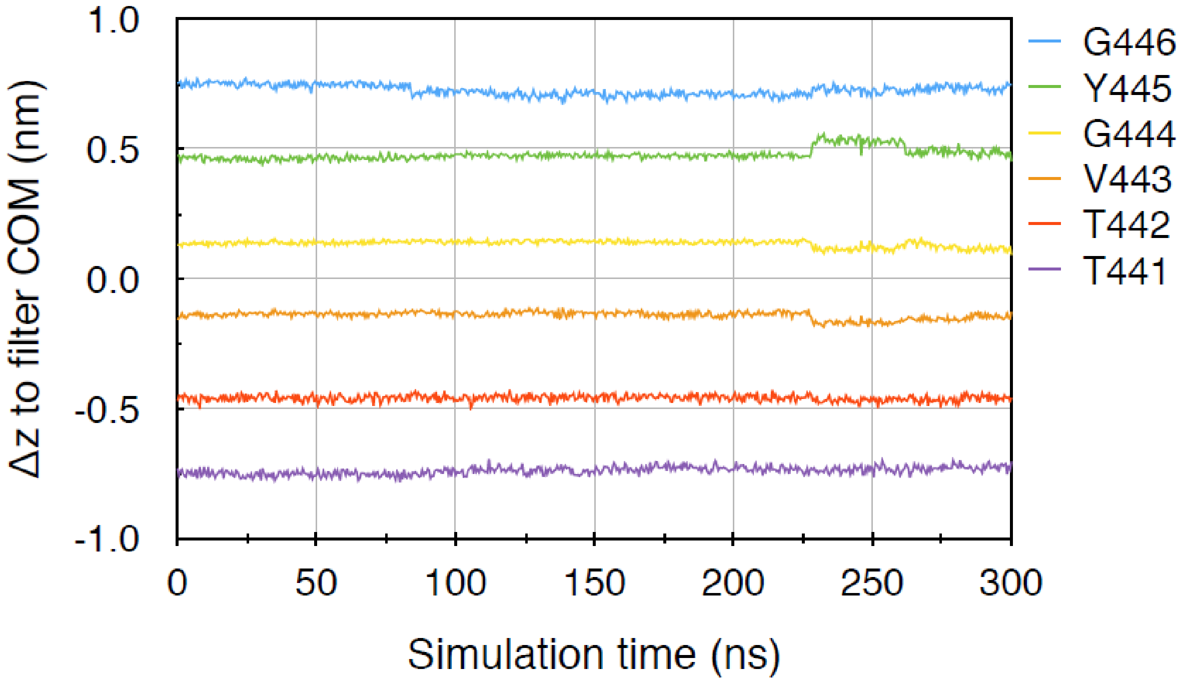

Supplementary figure S7. Distortion on the pore filter in molecular dynamics simulation. The increasing tension on the pore domain from the pulling has very little effect on the geometry of the pore, until it reaches the point where the potassium ion coordination is lost (229 ns). This is coupled with a vertical expansion on the pore, where in particular Y445 moves up while G444 and V443 move down, relative to the pore center-of-mass. The reorientation of the backbone carbonyl groups is one of the main reasons for the altered geometry. As the outward pulling motion continues the vertical positions of the pore residues relax back since there is no longer any ion bound in the outermost position to restrict the lateral distortion.

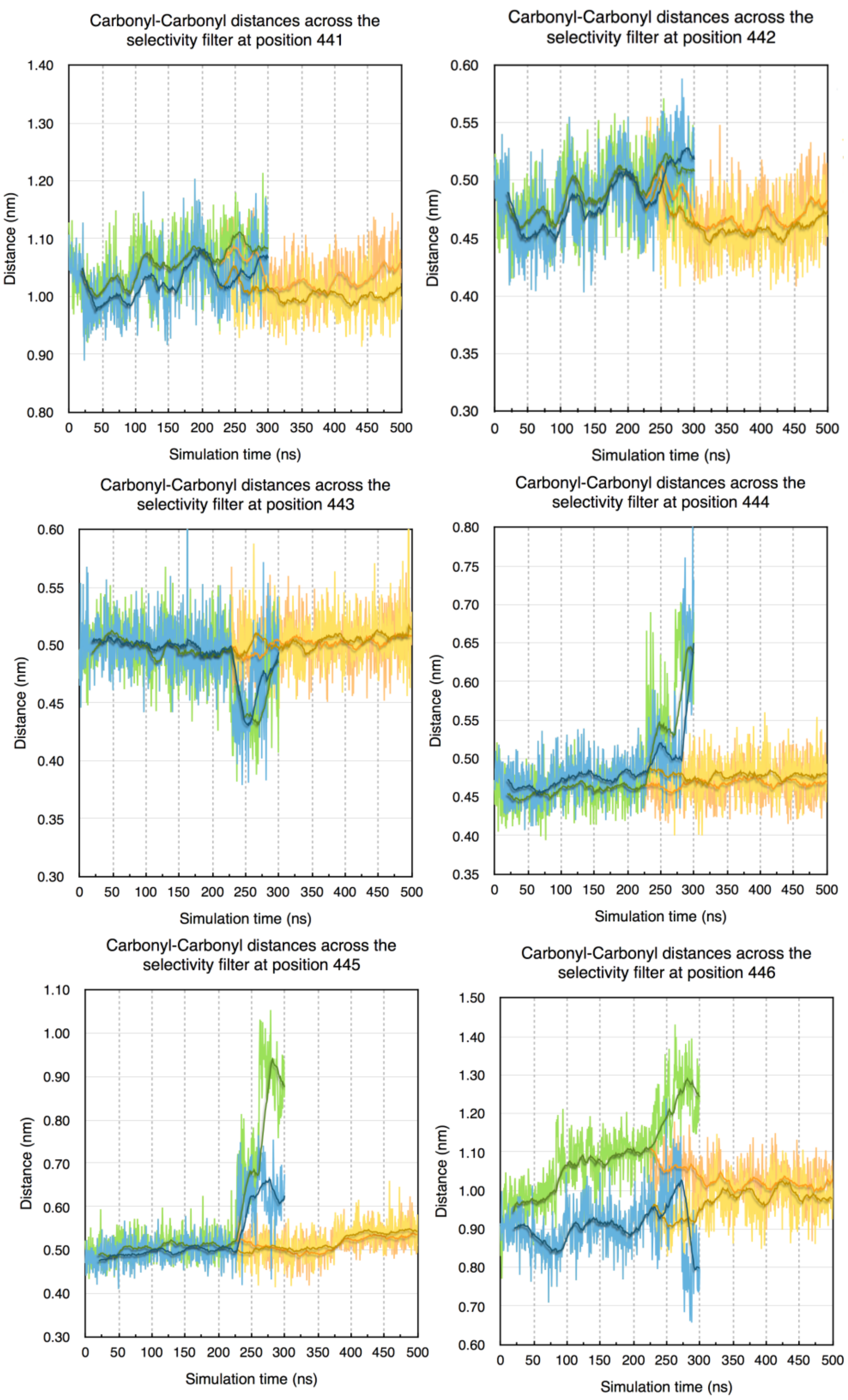

Supplementary figure S8: If the outward pulling of the F416 residues is stopped after 225 ns and the simulation continued without a force, the pore relaxes back roughly to its equilibrium geometry, and the ions remain bound. Blue/green colors indicate data for the two opposite domain pair distances in simulations with the force applied, while yellow/orange curves correspond to domain pair distances in simulations that were continued without the force applied.

SUPPLEMENTARY TABLES

Table I. Point mutations of F416 shifts the voltage dependence of the channel along the voltage axis. The midpoint values of the *G*(*V*) curves are shown below.

**Mutants [cells]**	**$V_{1/2}$ ± SD (mV)**
Wild type [3]	−19.0 ± 1.5
F416W [3]	−4.3 ± 3.8
F416A [3]	−9.0 ± 2.7
F416Q [3]	−3.7 ± 9.3
F416L [3]	8.3 ± 2.9
F416C [3]	−24.3 ± 5.1
F416E [3]	−11.3 ± 2.3
F416D [3]	5.5 ± 5.0
Wild type pH 6 [3]	−11.0 ± 1.0
F416H pH 6 [3]	8.0 ± 3.5
